# Loss of miR-101-3p Promotes Transmigration of Metastatic Breast Cancer Cells through the Brain Endothelium by Inducing COX-2/MMP1 Signaling

**DOI:** 10.3390/ph13070144

**Published:** 2020-07-07

**Authors:** Rania Harati, Mohammad G. Mohammad, Abdelaziz Tlili, Raafat A. El-Awady, Rifat Hamoudi

**Affiliations:** 1 Department of Pharmacy Practice and Pharmacotherapeutics, College of Pharmacy, University of Sharjah, Sharjah P.O. Box 27272, UAE; relawady@sharjah.ac.ae; 2 Sharjah Institute for Medical Research, University of Sharjah, Sharjah P.O. Box 27272, UAE; mmohd@sharjah.ac.ae (M.G.M.); rhamoudi@sharjah.ac.ae (R.H.); 3 Department of Medical Laboratories, College of Health Sciences, University of Sharjah, Sharjah P.O. Box 27272, UAE; 4 Department of Applied Biology, College of Sciences, University of Sharjah, Sharjah P.O. Box 27272, UAE; atlili@sharjah.ac.ae; 5 Clinical Sciences Department, College of Medicine, University of Sharjah, Sharjah P.O. Box 27272, UAE

**Keywords:** micro-RNA, breast cancer, brain metastasis, blood–brain barrier

## Abstract

Brain metastases represent one of the incurable end stages in breast cancer (BC). Developing effective or preventive treatments is hampered by a lack of knowledge on the molecular mechanisms driving brain metastasis. Transmigration of BC cells through the brain endothelium is a key event in the pathogenesis of brain metastasis. In this study, we identified miR-101-3p as a critical micro-RNA able to reduce transmigration of BC cells through the brain endothelium. Our results revealed that miR-101-3p expression is downregulated in brain metastatic BC cells compared to less invasive variants, and varies inversely compared to the brain metastatic propensity of BC cells. Using a loss-and-gain of function approach, we found that miR-101-3p downregulation increased transmigration of BC cells through the brain endothelium in vitro by inducing COX-2 expression in cancer cells, whereas ectopic restoration of miR-101-3p exerted a metastasis-reducing effect. In regulatory experiments, we found that miR-101-3p mediated its effect by modulating COX-2-MMP1 signaling capable of degrading the inter-endothelial junctions (claudin-5 and VE-cadherin), key components of the brain endothelium. These findings suggest that miR-101-3p plays a critical role in the transmigration of breast cancer cells through the brain endothelium by modulating the COX-2-MMP1 signaling and thus may serve as a therapeutic target that can be exploited to prevent or suppress brain metastasis in human breast cancer.

## 1. Introduction

Incurable, brain metastases (BM) remain a major cause of morbidity and mortality in breast cancer (BC) patients in spite of the major advances that have been made by systemic therapies. Current treatments are palliative with poor efficacy and include surgical resection, radiotherapy, chemotherapy and targeted therapies [1]. Most therapeutic drugs that are effective in treating primary tumors fail to treat brain metastases due to multiple factors including: 1) The blood–brain barrier that limits the ability of drugs to enter and evoke tumor cell death rendering the brain a “sanctuary site” [2], and 2) the acquired resistance properties of the metastatic cells caused by genetic and epigenetic alterations [1,3]. While available data indicate rising incidence as a consequence of improved systemic control and prolonged survival [4], finding effective and/or preventive treatments for breast cancer brain metastases (BCBM) is an urgent unmet medical need. However, the development of treatments is hampered by a lack of knowledge on the basic processes driving brain metastasis and colonization by cancer cells. A deeper understanding of BCBM biology and mechanisms will yield targets for effective or preventive strategies and will offer a great step forward in the treatment of BCBM [5]. 

Brain metastasis is a multi-step cascade that includes detachment of metastatic cells from the primary tumor, intravasation into the bloodstream or lymphatics, survival in the circulation, arrest into a capillary bed, attachment to the brain endothelium, extravasation through the Blood–Brain Barrier (BBB), survival in the brain microenvironment and brain colonization as a result of genetic predisposition and adaptation mechanisms [6,7,8,9,10,11,12]. One critical step in the establishment of BM is the transmigration of circulating metastatic cells through the BBB during extravasation [13,14]. The BBB is part of the neurovascular structure that separates the central nervous system (CNS) from the peripheral blood circulation. It is formed by the brain specialized microvascular endothelial cells tightly connected by tight and adherens junctions; pericytes; astrocyte; and neurons innervating the microvasculature. The barrier is essential for CNS homeostasis and limits entry of neurotoxic blood-borne molecules into the CNS [15,16]. During BM, circulating cancer cells acquire specific properties enabling them to adhere to the brain endothelial cells (BEC), induce alterations in the BBB components and micro-environment, transmigrate through the barrier and colonize the brain. Transmigration of tumor cells through the BBB is mediated by the upregulation of three genes: (HBEGF (Heparin-Binding Epidermal growth factor-like Growth Factor), COX-2 (PTGS2, Prostaglandin-Endoperoxide-Synthase-2), and ST6GALNAC5 (ST6-*N*-Acetylgalactosaminide-Alpha-2,6-Sialyltransferase-5)). HBEGF, a ligand for EGFR (Epidermal Growth Factor Receptor), was shown to increase invasiveness of cancer cells to the brain. COX-2, an enzyme upregulated by nuclear EGFR, synthesizes prostaglandins and promotes expression of MMP1 (matrix metalloproteinase-1) that degrades the intercellular junctions and permeabilizes the BBB. Lastly, ST6GALNAC5 was shown to promote BCBM by allowing adhesion and transmigration of cancer cells through the brain endothelium [12,17]. However, the genetic and epigenetic mechanisms by which cancer cells acquire their transmigratory capabilities allowing them to extravasate across the BBB are poorly characterized and remain among the most important challenges in breast cancer research. 

MiRs are a group of small non-coding RNAs that regulate gene expression at the post-transcriptional level by binding to target mRNAs and reducing their expression [18]. Dysregulation of miRs occurs in many types of cancer including BC, and is associated with tumorigenesis and drug resistance [19]. In the case of BC metastasis, a number of BM-specific miRNAs were shown to be differentially expressed between primary and brain metastatic tumors, suggesting a potential role of these microRNAs in the establishment of BCBM [19,20,21,22,23]. Particularly, miR-101-3p, a tumor-suppressive micro-RNA, was found to be downregulated in brain metastases compared to primary tumors in BC patients [24,25]. Downregulation of miR-101-3p was shown to be associated with poor prognosis, while miR-101-3p restoration inhibits proliferation, invasion, and lymph node metastasis of breast cancer cells [26,27] and promotes apoptosis [28]. However, the role of miR-101-3p in BCBM remains to be elucidated. On the basis of these observations, we conducted this work to investigate the role of miR-101-3p in transmigration of metastatic breast cancer cells through the brain endothelium, a key step of BCBM. 

## 2. Results

### 2.1. The Expression of mir-101-3p is Attenuated in Brain Metastatic Breast Cancer Cells 

**Figure 1:**
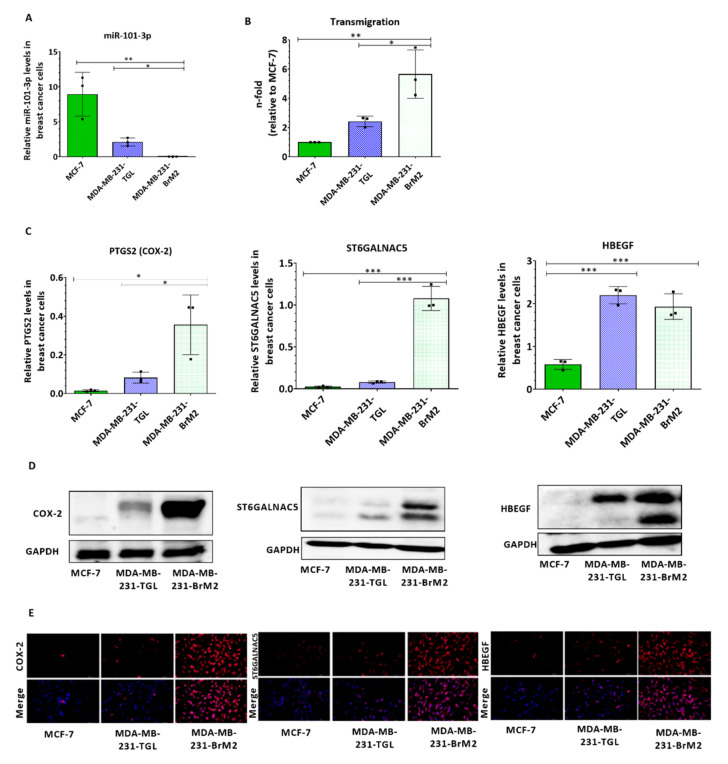
miR-101-3p levels are downregulated in brain metastatic breast cancer cells and vary inversely with their brain metastatic ability. (**A**) The expression profile of miR-101-3p was examined by real-time PCR in three breast cancer cell lines with different brain metastatic propensities (MCF-7, MDA-MB-231-TGL and MDA-MB-BrM2). Relative miR-101-3p level expression was normalized against the U6 small nuclear RNA levels. (**B**) The transmigration abilities of the different BC cells were examined by trans-endothelial migration assay. The amount of transmigrated cells was determined by fluorescence measurements.(**C**) The relative mRNA expression profile of three pro-metastasis genes known to mediate brain trans-endothelial migration of BC cells (PTGS2 coding for COX-2, ST6GALNAC5 and HBEGF) was examined in the three BC cell lines using real time PCR and normalized against GAPDH mRNA levels. (**D**,**E**) Western Blot analysis (**D**) and immunofluorescence images (**E**) of COX-2, ST6GALNAC5 and HBEGF in MCF-7, MDA-MB-231-TGL and MDA-MB-BrM2 cell lines. Experiments were carried out three times. Data are mean ±SD from three independent experiments. * *p* < 0.05, ** *p* < 0.01, *** *p* < 0.001.

To examine patterns of miR-101-3p expression in breast cancer cells, we used three Human BC cell lines with different metastatic propensity: 1) non-metastatic MCF-7; 2) metastatic MDA-MB-231-TGL (MDA231 for brevity); and 3) brain metastatic MDA-MB-231-BrM2 (MDA231Br for brevity), a brain-seeking variant of MDA231 generated by consecutive rounds of in vivo selection of clones that metastasized primarily to the brain in six to seven-week old beige nude and athymic mice injected intra-cardially with MDA231 [29]. Our results showed that endogenous expression levels of miR-101-3p were reduced in BC cells with higher brain metastatic capacity MDA231Br compared to the less invasive parental MDA231, while significant higher levels were detected in the non-metastatic MCF-7 cells (*p* = 0.0031) (Figure 1A). To assess whether a reduced miR-101-3p level correlate with the transmigration ability of BC cells, we examined the trans-endothelial migration ability of MCF-7, MDA231 and MDA231Br through a monolayer of human brain endothelial cell (HBEC) line (hCMEC/D3). hCMEC/D3 is a well-characterized human BEC line used to study the BBB in vitro as it retains the morphological characteristics of primary BEC and express a wide range of BBB structural (tight junctions, cell surface adhesion molecules) and functional (efflux transporters) components [30]. We found that MDA231Br are significantly more capable of penetrating the layer of brain endothelial cells than their corresponding parental cells (2.4-fold increase, *p* = 0.0201) and the non-metastatic MCF-7 (5.6-fold increase, *p* = 0.0034) (Figure 1B) suggesting that the transmigration ability of metastatic cells through the brain endothelium is inversely related to the expression of miR-101-3p. Additional statistical analysis demonstrated that miR-101-3p varies inversely with the trans-endothelial migration ability of BC cells (r = −0.8756, Table 1). To clarify the role of miR-101-3p in the transmigration of BC cells trough the brain endothelium, we compared the expression profile of miR-101-3p in BC cell lines with those of PTGS2 (coding for COX-2), ST6GALNAC5 and HBEGF, three pro-metastasis genes known to mediate the transmigration of tumor cells through the BBB [17]. As shown in Figure 1C, PTGS2 mRNA expression was 4- and 23-fold higher in MDA231Br compared to parental MDA231 (*p* = 0.0304) and non-metastatic MCF-7 (*p* = 0.0110) respectively, while a 14- and 50-fold increase of ST6GALNAC5 were measured in MDA231Br compared to parental MDA231 (*p* < 0.001) and MCF-7 (*p* < 0.001) respectively. However, no significant difference of HBEGF mRNA expression was noticed in MDA231Br compared to parental MDA231, with a three-fold increase compared to MCF-7 (*p* = 0.0008). Additional statistical analysis demonstrated that miR-101-3p expression varies inversely compared to mRNA expression of COX-2, ST6GALNAC5 and HBEGF (respectively, r = −0.8059; r = −0.7150; r = −0.9289; Table 1). Protein expression of the pro-metastasis genes was further examined in BC cells by western blot and immunofluorescence, and the results suggested that miR-101-3p expression was inversely related with COX-2, ST6GALNAC5 and HBEGF with highest expression of pro-metastasis genes detected in brain metastatic MDA231Br cells (Figure 1D,E). These findings suggest a potential role of miR-101-3p in transmigration of breast cancer cells through the brain endothelium.

### 2.2. Loss of miR-101-3p Promotes Trans-Endothelial Cell Migration by Upregulating COX-2/MMP-1 Signaling Pathway and Reducing Expression of the Inter-Endothelial Junctions

**Figure 2:**
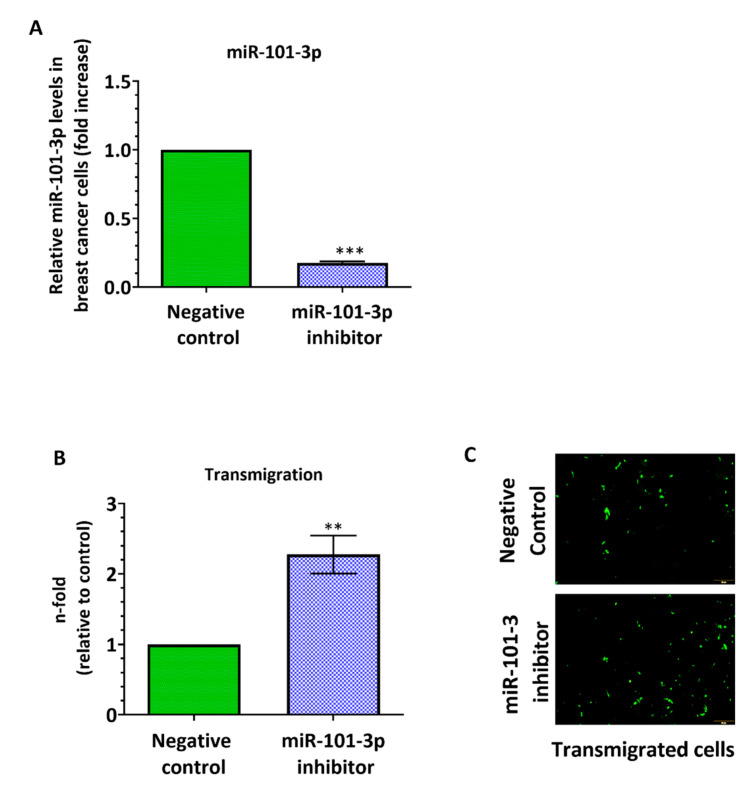
Ectopic downregulation of miR-101-3p in breast cancer cells promotes their transmigration through the brain endothelium. MDA-MB-231-TGL cells were transfected with miR-101-3p inhibitor (30 nM final concentration) or with negative control. (**A**) Relative miR-101-3p level expression was measured by real time PCR from total RNA. The small nuclear RNA U6 was used as internal control. (**B**) The transmigration abilities of the different BC cells were examined by trans-endothelial migration assay. (**C**) Representative images of transmigrated fluorescently labelled MDA-MB-231-TGL (small green cells) in control and anti-miR-101-3p transfected cells. Data are mean ±SD from three independent experiments. * *p* < 0.05, ** *p* < 0.01, *** *p* < 0.001.

To investigate the role of miR-101-3p in the trans-endothelial migration, we examined the effect of miR-101-3p suppression on the trans-endothelial cell migration ability of cancer cells. We downregulated the endogenous miR-101-3p expression in MDA231 cells using a mir-101-3p antagonist (anti-miR-101-3p), a chemically modified antisense single-stranded RNA that block the activity of endogenous miRs by complementarity. MDA231 transfection with anti-miR-101-3p significantly reduces miR-101-3p expression when compared to a negative control by 82% (Figure 2A) and this ectopic downregulation remarkably increased MDA231 transmigration through the hCMEC/D3 monolayer 2.3-fold (*p* = 0.0012) (Figure 2B,C).

**Figure 3:**
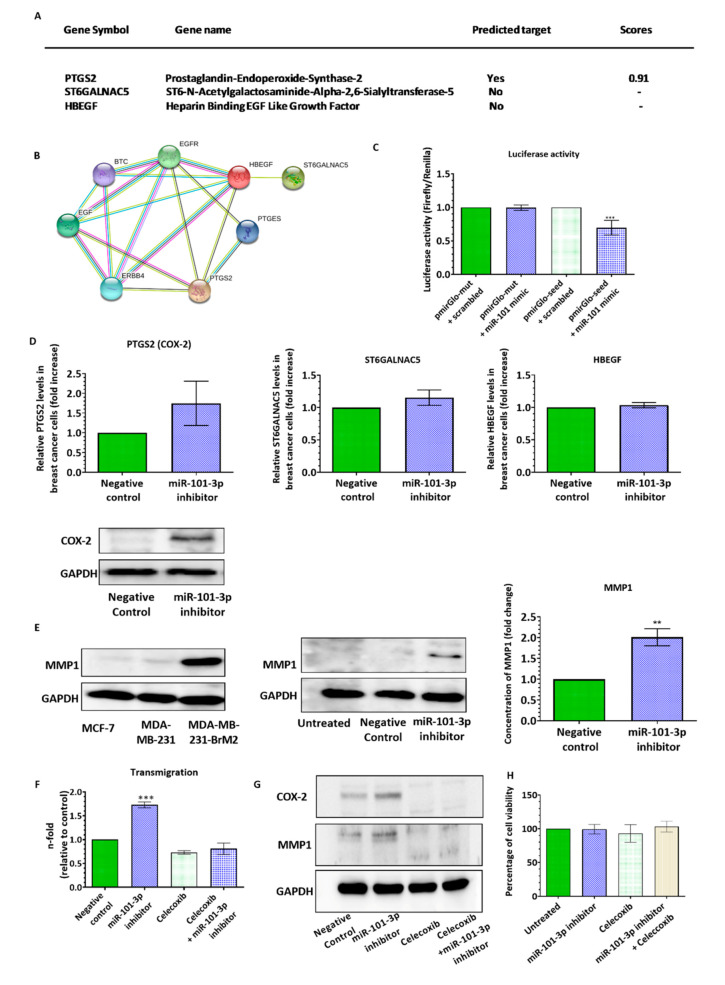
Ectopic downregulation of miR-101-3p in breast cancer cells promotes trans-endothelial migration through induction of COX-2-MMP1 expression. (**A**) List of has-miR-101-3p transendothelial migration-related predicted target genes in silico. (**B**) Protein–protein interaction network analysis retrieved from STRING (http://www.string-db.org/, version 9.1) showing the link between the different molecules involved in transmigration of breast cancer cells across the blood–brain barrier (BBB) (HBEGF, PTGS2 and ST6GALNAC5). Different colors of the lines represent the types of evidence for association: green line, neighborhood evidence; red line, fusion evidence; purple line, experimental evidence; light blue line, database evidence; black line, coexpression evidence; blue line, co-occurrence evidence; and yellow line, text-mining evidence. (**C**) Relative luciferase activity expressed as Firefly/Renilla luciferase activity. (**D**) Relative PTGS2 (COX2), ST6GALNAC5 and HBEGF mRNA and protein expression measured by real time PCR and western blot in control and anti-miR-101-3p transfected cells. (**E**) Western Blot analysis of MMP1 in the different BC cell lines and in control and anti-miR-101-3p transfected MDA-MB-231-TGL cells. MMP-1 levels released in the culture media were quantified by ELISA. (**F**) The transmigration ability of MDA-MB-231-TGL cells treated with miR-101-3p inhibitor and/or celecoxib was examined by trans-endothelial migration assay. (**G**) Western Blot analysis of COX-2 and MMP1 in MDA-MB-231-TGL cells treated with miR-101-3p inhibitor and/or celecoxib. (**H**) Cell viability of MDA-MB-231-TGL cells treated with miR-101-3p inhibitor and/or celecoxib was measured by MTT assay. Data are mean ±SD from three independent experiments. ** *p* < 0.01, *** *p* < 0.001.

To understand the molecular mechanisms underlying miR-101-3p effect on the trans-endothelial migration ability of BC cells, we searched for its potential pro-metastasis target genes using six prediction databases including mirTarBase 7.0, miRanda-mirSVR (microRNA.org), PicTar, miRDB, TargetScan 7.2 and TarBase v7.0 [31,32]. In our search, we focused on the genes involved in transmigration of BC through the brain endothelium, particularly, HBEGF, PTGS2 and ST6GALNAC5. The bioinformatics analysis identified PTGS2 as miR-101-3p target (score = 0.91), while ST6GALNAC5 and HBEGF were included due to their role in trans-endothelial migration (Figure 3A). Figure 3B was generated using STRING (version 9.1) with one recursive node network addition [33] and shows the interaction between these three molecules (HBEGF, PTGS2 and ST6GALNAC5). Interestingly, the STRING interaction shows an enrichment with epidermal growth factor (EGF) family proteins with four members present (EGF, BTC, ERBB4 and HBEGF). Using the Dual-Glo luciferase reporter assay, we demonstrated that the luciferase activity of the 3′UTR PTGS2 is reduced by mir-101-3p in MDA-MB-231 (*p* < 0.001), while co-transfection of miR-101-3p with a luciferase construct containing the mutant plasmid failed to decrease luciferase activity (Figure 3C). This in accordance with previous studies that showed that miR-101 interacts directly with the 3′-UTR of COX-2 mRNA leading to its post-transcriptional repression [34,35,36,37,38,39]. 

To further examine the effect of mR-101-3p on expression of pro-metastasis genes, we ectopically inhibited mir-101-3p expression in MDA231 by transfection followed by qPCR and Western blot analysis. As shown in Figure 3D, ectopic inhibition of mir-101-3p increases PTGS2 (COX-2) gene (1.74-fold) and protein expression with no effect on ST6GALNAC5 and HBEGF. These findings suggest that loss of miR-101-3p promotes trans-endothelial cell migration by inducing COX-2 expression. Previous reports showed that high COX-2 levels increase BC transmigration through the BBB by directly upregulating the expression of metalloproteinase-1 (MMP1) capable of increasing BBB permeability through degradation of inter-endothelial tight junctions (occludin and claudin-5), key components of the BBB [40]. To test whether loss of miR-101-3p increase trans-endothelial migration of BC cells by inducing COX-2/MMP1 pathway, we examined the effect of miR-101- downregulation on MMP1 expression and the Trans-Endothelial Electrical Resistance (TEER). We first measured MMP1 protein expression in BC cells and found that only MDA231Br cells express high level of MMP1, while no expression was detected in the non-metastatic MCF-7 and the less invasive MDA231 (Figure 3E), results that correlate with previous studies [40]. However, miR-101-3p ectopic downregulation in MDA231 increases the basal levels of MMP1 compared to the negative control), as well as levels of total MMP1 released in the culture media as measured by sandwich ELISA (Figure 3E). To confirm that miR-101-3p regulates the trans-endothelial migration through modulation of COX-2/MMP1 axis, we treated MDA231 cells with celecoxib (20 μM), a selective COX-2 inhibitor. Six hours later, cancer cells were transfected with miR-101-3p inhibitor (30 nM) and their transmigrative ability was assessed 48 h later. Our results showed that treatment of cancer cells with celecoxib and miR-101-3p inhibitor reduces COX-2 and MMP1 protein expression as well transmigration of BC cells through the brain endothelium compared to cells treated with miR-101-3p inhibitor alone (Figure 3F,G). As celecoxib was previously shown to reduce viability of BC cells at high doses (40 μM) [41], we performed an MTT assay to measure cell viability. Our results represented in Figure 3H showed that treatment of cancer cells with celecoxib at the dose of 20 μM and miR-101-3p inhibitor do not significantly reduces cell viability. Taken together, these data show that loss of miR-101-3p increases transmigration of BC cells through the brain endothelium by modulating COX-2/MMP1 axis.

**Figure 4:**
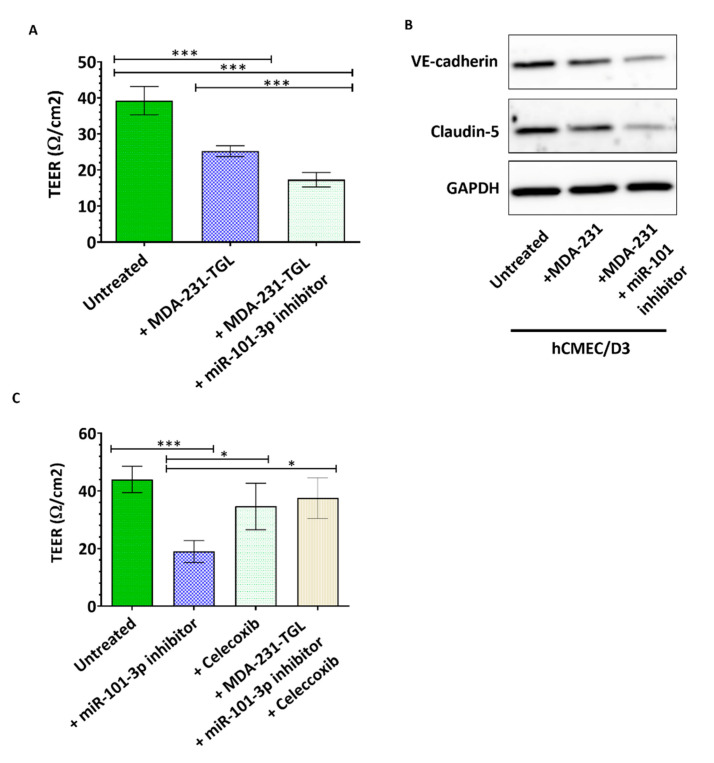
Ectopic downregulation of miR-101-3p in breast cancer cells interferes with brain intercellular junctions.(**A**,**C**) Transendothelial electrical resistance (TEER) was measured for brain endothelial cells co-cultured with MDA-MB-231-TGL cells treated with negative control, miR-101-3p inhibitor and/or celecoxib (**B**) Western Blot analysis of VE-cadherin and claudin-5 protein expression was measured by Western blot in hCMEC/D3 co-cultured with MDA-MB-231-TGL cells treated with miR-101-3p inhibitor or control. Data are mean ±SD from three to six independent experiments. * *p* < 0.05, *** *p* < 0.001.

We further investigated the effect of miR-101-3p downregulation on the permeability of hCMEC/D3 monolayer, and found that co-culture of hCMEC/D3 with untransfected MDA231 reduced the monolayer TEER by 36% (*p* < 0.001) while co-culture with MDA231 cells pretreated with anti-miR-101-3p further reduced the TEER to 56% (*p* < 0.001) (Figure 4A). We also examined expression of two inter-endothelial junctional proteins, VE-cadherin and claudin-5 in hCMEC/D3 when co-cultured with untransfected MDA231 or MDA231 pretransfected with anti-miR-101-3p. VE-cadherin and claudin-5 are key determinants of the barrier permeability [42,43] and our results show that MDA-231 reduces VE-cadherin and claudin-5 protein expression in hCMEC/D3, and this expression was further reduced to undetectable levels in hCMEC/D3 when co-cultured with MDA231 pretransfected with anti-miR-101-3p (Figure 4B). Interestingly, co-culture of hCMEC/D3 with MDA231 cells pretreated with anti-miR-101-3p and celecoxib did not affect the barrier TEER (Figure 4C). These results show that loss of miR-101-3p in BC cells increase their migration through the brain endothelium by inducing COX-2/MMP1 expression which in turn decreases expression of inter-endothelial junctions. 

### 2.3. miR-101-3p Reduces Trans-Endothelial Cell Migration by Reducing COX-2/MMP-1 Expression and Protecting the Endthelial Barrier

**Figure 5:**
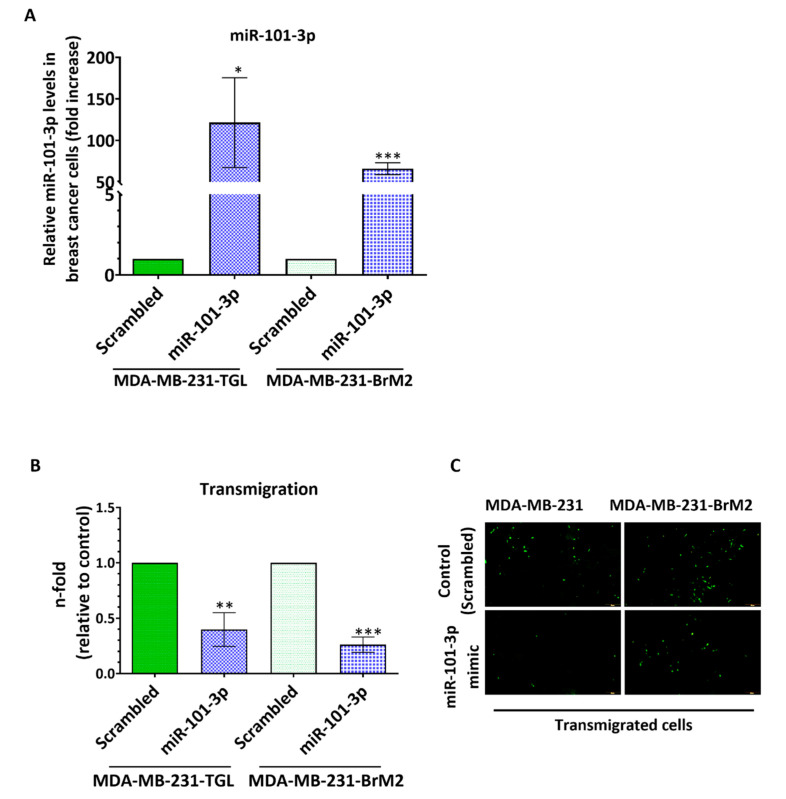
Ectopic upregulation of miR-101-3p in low expressing breast cancer cells reduces BC trans-endothelial migration.MDA-MB-231-TGL and MDA-MB-231-BrM2 cells were transfected with miR-101-3p mimic (5 nM final concentration) or with scrambled control. (**A**) Relative miR-101-3p level expression was measured by real time PCR from total RNA. The small nuclear RNA U6 was used as internal control. (**B**) The transmigration abilities of the different BC cells were examined by the trans-endothelial migration assay. (**C**) Representative images of transmigrated fluorescently labelled MDA-MB-231-TGL and MDA-MB-231-BrM2 (small green cells) in control and miR-101-3p mimic transfected cells. * *p* < 0.05, ** *p* < 0.01, *** *p* < 0.001.

Our data indicates that loss of miR-101-3p enhances trans-endothelial cell migration through upregulation of COX-2/MMP-1 signaling pathway. This finding suggests that miR-101-3p can be exploited therapeutically to prevent brain metastasis. To evaluate its transmigration-suppressing effect, we ectopically increased miR-101-3p endogenous levels in MDA231 and MDA231Br cells by transfection and examined the resulting effect on the trans-endothelial cell migration ability of cancer cells. miR-101-3p endogenous levels were induced with miR-101-3p mimic, a synthetic double-stranded RNA that stimulates naturally occurring mature miR-101-3p. Transfection with miR-101-3p significantly increases miR-101-3p expression in MDA231 and MDA231Br compared to the control (scrambled miR) (Figure 5A). Increased miR-101-3p remarkably reduced the transmigratory ability of MDA231 and MDA231Br by 60% (*p* = 0.0024) and 74% (*p* < 0.001) respectively (Figure 5B,C).

**Figure 6:**
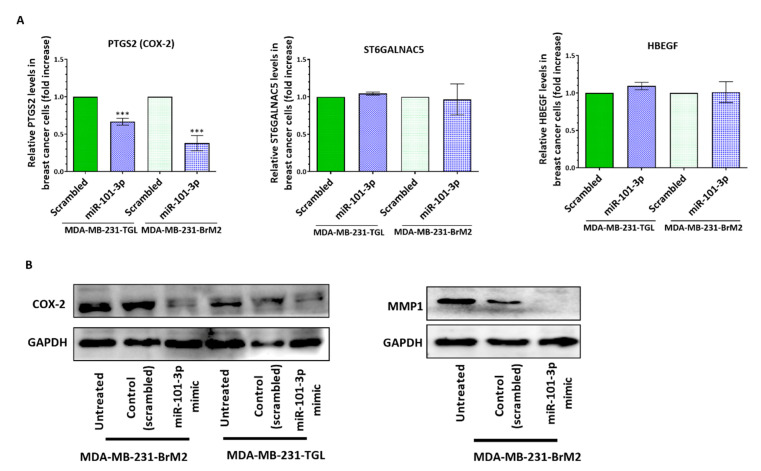
Ectopic upregulation of miR-101-3p in breast cancer cells attenuates their transmigration through the brain endothelium by reducing COX-2-MMP1 expression.MDA-MB-231-TGL and MDA-MB-231-BrM2 cells were transfected with miR-101-3p mimic (5 nM final concentration) or with scrambled control. (**A**) Relative PTGS2, ST6GALNAC5 and HBEGF mRNA measured by real time PCR in control and miR-101-3p mimic transfected cells. (**B**) Western Blot analysis of COX-2 and MMP1 in control and anti-miR-101-3p transfected MDA-MB-BrM2 cells. Data are mean ±SD from three independent experiments. *** *p* < 0.001.

To further demonstrate miR-101-3p contribution to the considerable reduction of BC cells transmigration through human brain endothelial cells, we assessed the effect of miR-101-3p upregulation on the pro-metastasis gene expression. Our results represented in Figure 6A showed that miR-101-3p reduces gene expression of COX-2 in MDA231 (*p* = 0.0002) and MDA231Br (*p* = 0.0005), while no changes were observed for ST6GALNAC5 and HEBGF. Interestingly, miR-101-3p upregulation reduced protein expression of COX-2 and MMP-1 in MDA231Br (Figure 6B), which shows that restoring miR-101-3p in breast cancer cells reduces their transmigration through the brain endothelium by reducing COX-2/MMP1 expression.

**Figure 7:**
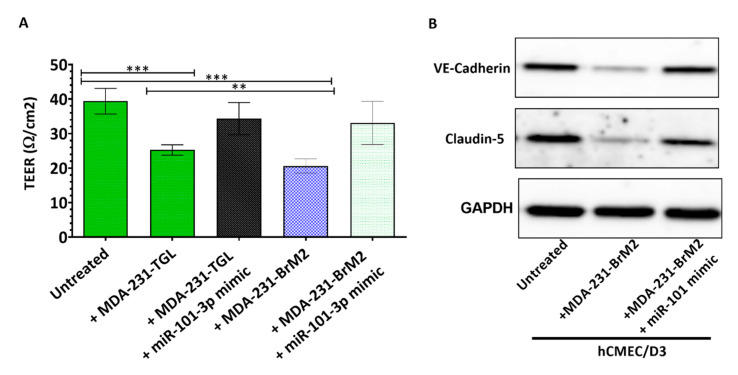
Ectopic upregulation of miR-101-3p in breast cancer cells preserves the brain endothelial inter-cellular junctions. (**A**) Transendothelial electrical resistance (TEER) was measured for brain endothelial cells co-cultured with MDA-MB-231-TGL and MDA-MB-BrM2 cells treated with miR-101-3p mimic or controls. (**B**) Western Blot analysis of VE-cadherin and claudin-5 protein expression was measured by Western blot in hCMEC/D3 co-cultured with MDA-MB-231-TGL and MDA-MB-BrM2 cells treated with miR-101-3p mimic or controls. Data are mean ±SD from three to six independent experiments. ** *p* < 0.01, *** *p* < 0.001.

**Figure 8:**
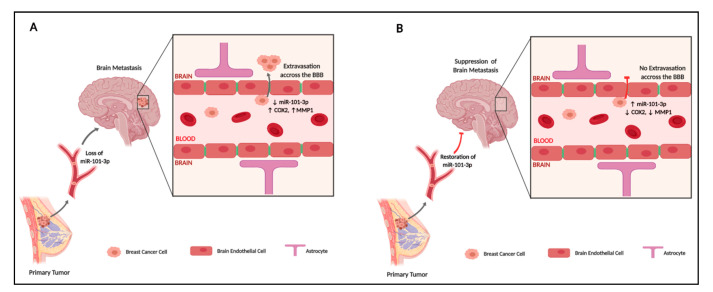
A proposed model for the pathological role of miR-101-3p in transmigration of metastatic breast cancer cells through the brain endothelium (created with BioRender).

We further investigated the effect of miR-101-3p upregulation on the permeability of hCMEC/D3 monolayer, and found that co-culture of hCMEC/D3 with untransfected MDA231 and MDA231Br reduced the monolayer TEER by 36% (*p* < 0.001) and 48% (*p* < 0.001) respectively, while no significant changes in TEER were observed when MDA231 and MDA231Br were pretransfected with miR-101-3p mimic (Figure 7A). We also examined expression of VE-cadherin and claudin-5 in hCMEC/D3 when co-cultured with untransfected MDA231 or MDA231 pretransfected with miR-101-3p mimic and found that MDA-231 reduces VE-cadherin and claudin-5 protein expression in hCMEC/D3. However, VE-cadherin and claudin-5 expression remained similar to the control when MDA231 and MDA231Br were pretransfected with miR-101-3p mimic (Figure 7B). These results show that inducing miR-101-3p in BC cells reduces their migration through the brain endothelium by reducing COX-2/MMP1 expression which in turn protects the brain endothelium (Figure 8). 

## 3. Discussion

In this study, we provide first-time evidence that miR-101-3p regulates the transmigration of breast cancer cells through the brain endothelium, a key step in brain metastasis. We found that miR-101-3p expression is lost in brain metastatic breast cancer cells, while ectopic expression of miR-101-3p reduces transmigration of cancer cells through the brain endothelium through reduction of COX-2/MMP1 expression which interferes with the inter-endothelial junctions. Our findings support the notion that BCBM cascade is regulated by micro-RNAs that could be exploited therapeutically to suppress or prevent BM of BC cells.

Brain metastasis is usually a late event in breast cancer, but its incidence is rising as a consequence of effective systemic therapies and prolonged life span. Once diagnosed with BM, the median survival of treated patients is four to six months with available treatments that are palliative in the majority of cases [1]. The limited cerebral efficacy and poor prognosis with the available conventional therapies underscore the need for better therapeutic approaches, particularly novel therapies that focus on preventing brain metastases. However, for such preventive strategies to become within reach, further research is needed to elucidate the molecular basis driving brain metastasis. 

In recent years, micro-RNAs, a class of non-coding RNAs, have emerged as attractive tools and targets for novel anti-cancer therapeutic approaches. Since their discovery, micro-RNAs have been shown to regulate a wide range of biological processes including carcinogenesis, where micro-RNAs were shown to be heavily dysregulated in many cases of cancer [42] with miRNAs acting as oncomiRs or tumor suppressors. Interestingly, molecules targeting miRNAs have shown promise in preclinical studies, and several miRNA-based therapeutics have reached clinical development [19]. For instance, MRX34 is a liposome-formulated double-stranded mimic of tumor suppressor microRNA-34 (miR-34) has reached phase I clinical trials for treating unresectable primary liver cancer [44], and Miravirsen, a miR-122 inhibitor, has reached phase II trials for treating hepatitis [45].

In the case of breast cancer, a growing number of studies have highlighted the role of microRNAs in the pathogenesis of BM and their therapeutic potential [46,47]. For instance, Zhang et al. showed that miRNA-1258 suppresses BC brain metastasis by downregulating the expression and activity of heparanase, an enzyme with pro-tumorigenic, pro-angiogenic and pro-metastatic properties [48]. Xing et al. showed that miRNA-509 restoration significantly suppresses the ability of cancer cells to metastasize to the brain by modulating RhoC/MMP9 and TNFα that affect cancer cells invasion and BBB permeability, respectively [25]. On the other hand, Tominaga et al. showed that brain metastatic cancer cells promote BCBM by releasing microRNA-181c-containing extracellular vesicles capable of destructing the BBB [49]. These observations point to miRNA as promising therapeutic targets, however, the mechanistic roles of microRNAs in regulating brain metastasis of breast cancer cells remain largely unknown and require further research to come a panel of specific micro-RNA profiles that regulate the different steps of breast cancer brain metastatic cascade and that are suitable as therapeutic agents to suppress or prevent BM.

In the present study, we identified miR-101-3p as a metastasis regulator micro-RNA that exerts its role by reducing COX-2/MMP1 expression and subsequently reduces transmigration of BC cells through the brain endothelium. The role of miR-101-3p in tumorigenesis and metastasis was previously established [24,26,27,28]. For instance, He et al. showed that miR-101 levels in gastric cancer tissues were downregulated compared to normal tissue, and lower levels of miR-101 were associated with increased tumor invasion and lymph node metastasis. Overexpression of miR-101 in gastric cancer cell lines inhibited cell proliferation and induced apoptosis in vitro and inhibited tumor growth in vivo through downregulation of COX-2 [50]. Similarly, Shao et al. showed that miR-101 is downregulated in esophageal squamous cell carcinoma (ESCC), while transfection of miR-101 in ESCC cell lines suppressed cell proliferation, migration and invasion through modulation of COX-2 expression [38]. Other studies also showed that downregulation of miR-101 is involved in the initiation and development of glioma via COX-2 [34], while exogenous miR-101 is able to suppress proliferation and growth of prostate cancer cells in vitro and in vivo [37]. In breast carcinogenesis, miR-101 was shown to be downregulated in different subtypes of human breast cancer tissues, and this downregulation was linked to increased cellular proliferation and invasiveness via targeting Stathmin1 [26]. Another study showed that miR-101 suppressed proliferation and promoted apoptosis in breast cancer cells by targeting Jak2 [28]. In the case of BCBM, miR-101-3p expression was found to be downregulated in breast cancer patients with brain metastasis [25], however, its specific role in BCBM remains to be elucidated.

Our results show that miR-101 exerts an anti-metastatic effect by reducing COX-2, a key regulator of inflammation-producing prostaglandins, often found to be overexpressed in tumor tissues. COX-2 is also known to act as a key gene that mediates transmigration of BC cells through the BBB during BM, along with two other genes HEBGF and ST6GALNAC5 [17,34,36,51]. Specifically, COX-2 is upregulated by nuclear EGFR and synthesizes prostaglandins to directly upregulate the expression of matrix metalloproteinase-1 (MMP1) which in turn degrades the inter-endothelial tight junctions, occludin and claudin, increases the BBB permeability and promotes transendothelial migration of BC cells and brain metastasis [40]. Our results showed that the loss of miR-101-3p upregulates COX-2 and induces MMP1 expression. Increased COX-2/MMP1 was associated with loss of claudin-5 and VE-cadherin, two key components of the inter-endothelial junctions. Claudin-5 is a key component of the brain endothelial tight junctions and a key determinant of the BBB permeability. It selectively decreases the permeability to small molecules and its loss was previously shown to reduce trans-endothelial resistance [52]. Dysregulation of claudin-5 and changes in the endothelial permeability were demonstrated in a number of pathological processes, including stroke, inflammation, and brain tumors [42]. Additionally, it was demonstrated that claudin5 is degraded by matrix metalloproteinases which increases BBB permeability [53]. VE-cadherin is another vital component of the BBB inter-endothelial junctions, responsible for the assembly and maintenance of adherens junctions, as well as regulation of vascular barrier integrity. Deletion of VE-cadherin in mice was shown to disrupt and alter localization of tight junctions [43]. On the other hand, VE-cadherin was shown to be a direct target of miR-101 in the tumor cells [54] and the brain endothelium [55], and expression level of claudin-5 was found to be governed by that of VE-cadherin. However, our results showed no changes in VE-cadherin and claudin-5 expression in the brain endothelium when co-cultured with brain metastatatic cells pre-transfected with miR-101mimic which suggests that miR-101 was not transferred to the endothelial cells. Taken together, our results showed that induction of COX-2 and MMP1 in BC cancer cells was associated with loss of claudin-5 and VE-cadherin in the brain endothelial cells and increased trans-endothelial migration.

During the last decade, numerous groups studied the anti-cancer action of COX-2 inhibitors. It was suggested that synthetic cyclooxygenase-2 inhibitors may hold promise for cancer chemoprevention; however, toxicity problems suggest that new strategies are needed, while a better understanding of the mechanisms regulating COX-2 will open avenues and yield targets for BM therapeutic and preventive strategies. Our results suggest that miR-101-3p is a potential target that can be used to modulate COX-2/MMP1 signaling and reduces trans-endothelial migration of BC cells.

Two other genes that mediate transmigration of metastatic BC cells through the brain endothelium are HBEGF and ST6GALNAC5. HBEGF is a ligand for EGFR that was shown to increase migration and invasiveness of cancer cells and was identified specifically in brain metastasis compared to the bone and lung. ST6GALNAC5 is specific to brain metastatic cancer cells and was shown to allow cell–cell interactions and to aid in adhesion and transmigration of cancer cells through the brain endothelium. Furthermore, the knockdown of ST6GALNAC5 and inhibition of EGFR with cetuximab was shown to decrease brain metastasis [17,56]. Our results show that restoring miR-101-3p expression in BC cells reduces transmigration of breast cancer cells through the brain endothelium by downregulating COX-2/MMP1 signaling, however without clear effect on HBEGF and ST6AGLNAC5 expression. This could explain the lack of total inhibition of trans-endothelial migration in our study and suggest that a better understanding of the interaction between COX-2, HBEGF and ST6GALNAC5, as well as a better characterization of the complex cellular and molecular mechanisms that govern cancer cells adhesion and transmigration through the BBB are needed to optimize the therapeutic strategies that aim at suppressing breast cancer brain metastasis by inhibiting the migration of cancer cells through the brain endothelium.

In conclusion, our study demonstrates the role of miR-101-3p in controlling transmigration of breast cancer cells through the brain endothelium, a key step in BCBM. Our results show that the exogenous modulation of miR-101-3p attenuates the trans-endothelial migration of metastatic breast cancer cells in vitro through reduction of COX-2/MMP1 signaling. These results imply that miR-101-3p is a potential target that can be exploited therapeutically to suppress or prevent breast cancer brain metastasis that is, to date, incurable.

## 4. Materials and Methods 

### 4.1. Cells and Cell Culture

MCF-7 and MDA-MB-231 breast cancer cell lines were purchased from ATCC. The brain metastatic MDA-MB-231-BrM2 and the parental cells MDA-MB-231-TGL were obtained from Dr Joan Massagué (Memorial Sloan-Kettering Cancer Center, New York, NY, USA) [17,29]. Cancer cells were cultured in DMEM medium supplemented with 10% FBS, 1X L-Glutamine and antibiotics (1000 U Penicillin/1000 U Streptomycin). The immortalized human cerebral microvascular endothelial cells (hCMEC/D3) were obtained from Cedarlane (Tebu-Bio, France) and cultured in EndoGRO™-MV Complete Medium (cat# SCME004, EMD Millipore, USA) supplemented with 1ng/mL FGF-2 (cat# GF003, EMD Millipore, USA) and antibiotics. hCMEC/D3 were grown to confluence on tissue flasks precoated with thin collagen I coating (08-115, EMD Millipore) as recommended by the manufacturer. All cells were maintained in a 95% humidified air and 5% CO2 incubator at 37 °C.

### 4.2. Establishment of the In Vitro Brain Endothelial Cells (BEC) Monolayer

hCMEC/D3 (5 × 10^4^ cells) were plated on the upper side of a collagen I-precoated Transwell membrane (pore size 0.4 μm, growth area 1.12 cm2) in the endothelial medium. Under these experimental conditions, hCMEC/D3 cells form a confluent and tight monolayer within 72 h with the highest Transendothelial electrical resistance (TERR) values between days 3 and 7.

### 4.3. Transendothelial Electrical Resistance (TEER) Measurement

Integrity of the in vitro BBB model was monitored by measuring the TEER using an Endohm 12 chamber and an Endohmeter EVOMX (World Precision Instruments) following the manufacturer’s instructions.

### 4.4. In Silico Bioinformatics Analysis

In silico bioinformatics analysis was carried out to identify miR-101 target genes that are involved in transmigration of cancer cells through the brain endothelium. Initially, we searched for the targets of miR-101-3p using 6 prediction databases including mirTarBase 7.0, miRanda-mirSVR (microRNA.org), PicTar, miRDB, TargetScan 7.2 and TarBase v7.0 [31]. The target genes identified in more than 3 databases were retained. The filtered set was then annotated by adding genes with similar function, and the interaction between them was assessed using STRING (version 9.1) [33].

### 4.5. MicroRNA Transfection 

Breast cancer cells were cultured to ~ 70% confluence. For inhibition studies, 30 nM of miR-101-3p inhibitor (Anti-hsa-miR-101-3p miScript miRNA Inhibitor, cat# 219300) or its negative control (miScript Inhibitor Negative Control, cat# 1027271, Qiagen) were transfected using HiPerFect Transfection Agent in culture media for 48h. Down- and upregulation of miR-101-3p were assessed by QPCR. To introduce mir-101-3p mimic, cells were transfected with either 5nM of miR-101-3p mimic (Syn-hsa-miR-101-3p miScript miRNA Mimic, Cat# 219600, MIMAT0000099: 5’UACAGUACUGUGAUAACUGAA, Qiagen, Germany), or its negative (scrambled) control (AllStars Negative Control, cat# SI03650318, Qiagen), using HiPerFect Transfection Agent (Qiagen, Germany) in culture media for 48h. 

### 4.6. Pharmacological Treatment and Cell Viability Assay

Parental MDA-MB-231 cells were seeded at a concentration of 5 × 10^3^ cell/well in 96-well plate. After 24 h, cells were treated with 20 μM of celecoxib (ab141988, abcam). Six hours later, cells were transfected with miR-101-3p inhibitor. MTT (3-(4,5-dimethylthiazol-2-yl)-2,5-diphenyltetrazolium bromide) assay was performed after 48h; 10 μL MTT (5 mg/mL) was added and the cells were incubated for 4 h. Supernatant was then removed and 50 μL of DMSO (dimethyl sulfoxide) was added to each well. Cell viability was assessed by measuring the absorbance at 540 nm wavelength using MultiskanGo microplate spectrophotometer (Thermofischer, Waltham, MA, USA).

### 4.7. Real-Time PCR

Kits and reagents used for real-time PCR were from Qiagen, Germany. MiRNAs and total RNAs were extracted from cells using the miRNeasy Micro Kit (cat# 217084). First strand cDNA synthesis and amplification were performed using the miScript II RT Kit (cat# 218161) for miRNA and the RT2 First Strand Kit (Cat#, 330401) for mRNA. SYBR Green quantitative PCR amplifications were performed on the “Applied Biosystems^®^ StepOne™ Real-Time PCR System” using the miScript SYBR Green PCR Kit (Cat# 218075) for miRNA expression and the RT^2^ SYBR Green ROX qPCR Mastermix (cat# 330522) for mRNA expression following the manufacturer’s protocol. The following primers from Qiagen were used: Hs_miR-101_3 miScript Primer Assay (Cat# MS8300072), RT^2^ qPCR Primer Assay for Human PTGS2 (cat# PPH01136F), RT^2^ qPCR Primer Assay for Human ST6GALNAC5 (cat# PPH14652A), RT^2^ qPCR Primer Assay for Human HBEGF (cat# PPH02589A). Hs_RNU6-2_11 miScript Primer Assay (cat# MS00033740) and RT^2^ qPCR Primer Assay for Human GAPDH (Cat# PPH00150F) were used as an internal control. PCR efficiency was checked prior to the analysis by performing a dilution series experiment using each target assay.

### 4.8. Cancer Cells Trans-Endothelial Migration Assay 

For trans-endothelial migration assay, the hCMEC/D3 monolayer was cultured on 3 μm pore filter inserts in phenol red free endothelial medium (described above). On the day of the experiment, TEER of the monolayer was monitored. Chambers with a TEER lower than 35 ohm/cm2 were discarded. Transfected or control BC cells were then labeled using CellTracker™Green CMFDA fluorescent dye (cat# C2925, Thermofischer-Scientific, USA) following the manufacturer’s recommendations. A total of 2 × 104 fluorescent tumor cells were added gently to the hCMEC/D3 monolayer and left to transmigrate to the lower chamber for 24 h at 37 °C. After 24 h, the upper chambers were washed with PBS and scrapped gently with cotton wool. Tumor cells in the basal compartment that have transmigrated through the BEC were lysed using RIPA 1X buffer and fluorescence in the lower compartment was quantified in a fluorescent microplate reader at wavelength excitation/emission: 492/517 nm.

### 4.9. Western Blot

Western blot analysis was performed according to standard procedures. Briefly, cells were lysed into a lysis solution containing RIPA lysis buffer 1X and a cocktail of inhibitors (1 mM PMSF, 1 mM sodium orthovanadate, 1 μg/mL leupeptin, and a protease inhibitor cocktail (cat# P2714, Sigma-Aldrich, Germany). After lysis, the amount of proteins was determined using the Thermo Scientific Pierce BCA Protein Assay Kit. The cell lysates were diluted in 1X Laemelli’s buffer solution, at 95 °C for 5 min. Total protein (10 μg/lane) were separated on 12% SDS-polyacrylamide gels and then transferred onto nitrocellulose membranes (Amersham, Germany) by the wet-transfer system (Bio-Rad, USA). The membranes were blocked with 5% skim milk in Tween Tris-Buffered saline (TTBS) for 1h and incubated with primary antibodies at 4 °C overnight and then with secondary anti-rabbit IgG (1/3000) or anti-mouse IgG (1/3000) antibodies conjugated to horseradish peroxidase for 1 h at room temperature. The immunoblots were then visualized with enhanced chemiluminescence (ECL) kit (using the *ECL™ Prime Western Blotting* System GE Healthcare, cat# RPN2232 or the Bio-Rad Clarity Western ECL Substrate, cat# 1705061) and imaged using ChemiDoc™ Touch Imaging System (Bio-Rad). The following primary antibodies from Abcam and Santa Cruz Biotechnology were used: Anti-COX-2 (ab15191) 1/1000; Anti-ST6GALNAC5 (ab201575) 1/400; Anti-HB-EGF (sc-74526) 1/250; Anti-MMP1 (ab38929) 1/5000, Anti-Claudin 5 (ab15106) 1/3000, Anti-VE Cadherin (ab33168) 1/1000 and anti-GAPDH (ab9485) 1/7500 as loading and transfer control.

### 4.10. Immunofluorescence

Breast cancer cells were cultured on glass coverslips. For immunostaining, cells were fixed with 4% paraformaldehyde for 10 min, permeabilized with 0.1% Triton™ X-100 in PBS for 10 min, blocked with 2% BSA for 1h, and then incubated with the primary antibodies anti-COX-2 (ab15191) 1/500; anti-ST6GALNAC5 (ab201575) 1/200; anti-HB-EGF (sc-74526) 1/200 at 4 °C overnight. Cells were washed with PBS and incubated for 1h at room temperature with secondary antibodies donkey anti-mouse IgG H&L (Phycoerythrin) (ab7003) and donkey F(ab’)2 anti-rabbit IgG H&L (PE) (ab7007). Samples were them mounted in ProLong^®^ Gold Antifade Reagent with DAPI (cat# 8961, Cell Signaling, USA) and examined with Olympus BX43 fluorescent microscope.

### 4.11. Luciferase Assay

For miR target validation, the putative (seed) binding site of miR-101-3p in the 3′UTR of COX-2 predicted by TargetScan 7.2 (position 1737-1744 of PTGS2 3’ UTR) and the mutant (MUT) 3′UTR of COX-2 with one nucleotide substitution were cloned into pmirGLO Dual-Luciferase miRNA Target Expression Vector (pmirGLO-empty, Promega, USA) downstream of the firefly luciferase gene (XbaI and Nhe sites) to obtain Luc Reporter Construct (pmirGLO-seed and pmirGLO-mut). The primers used for cloning 172 bp of 3′-UTR-COX2 in pmirGLO vector: Forward 5′AAGCTAGCTGATATCTAAGTAGTTCTCAGC 3′; Reverse 5′AATCTAGACAATGATTGTAGGCTTAAACAC 3′ (seed). The mutant primers used to introduce a mutation by site directed mutgenesis (GeneArt Site-Directed Mutagenesis, invitrogen, USA) are the following: Forward 5′ ATTTAATGGTATTGTATATTACTTA 3′; Reverse 5′ TAAGTAATATACAATACCATTAAAT 3′ (mut). MDA-MB-231 cells were plated at a density of 10^5^ cells/well in a 96-well plate and co-transfected with pmirGLO-empty (100 ng), pmirGLO -seed (100 ng), miR-101 mimic (5 nM) and negative scrambled control depending on treatments and following Lipofectamine 3000 reagent protocol. Cells were harvested 24 h after transfection and cell lysates were used for Dual-Luciferase^®^ Reporter Assay System analysis according to the manufacturer’s instructions (Promega, USA).

### 4.12. Enzyme-Linked Immunosorbant Assay (ELISA)

MMP-1 protein levels in cell culture supernatants were quantified by ELISA using the Human Total MMP-1 DuoSet (cat# DY901B, R&D Systems) according to manufacturer’s instructions. The detection limit was 62.5 pg/mL. Signal saturation was observed at concentrations > 4000 pg/mL. The optical density was measured using a Varioskan Flash multimode reader (Thermo Scientific, USA) at a wavelength of 450 nm. Averages of the optical densities obtained from the duplicate readings for each standard, control, and sample were calculated. MMP-1 concentrations were then calculated by generating a four-parameter logistic (4-PL) curve-fit. 

### 4.13. Statistical Analysis

Results are expressed as mean ± SD (standard deviation of the mean) of three independent experiments with (2-3) replicates each. *P*-values were calculated using independent unpaired Student’s t tests (parametric) or one-way ANOVA (analysis of variance) followed by Bonferroni post hoc test for multiple comparisons. Differences were considered statistically significant at probability levels of *p* < 0.05(*), *p* < 0.01(**), and *p* < 0.001(***). Calculations and figures were performed using the statistical software GraphPad-Prism 8.2.0.

## Figures and Tables

**Table 1:** Correlation of miR-101-3p expression with PTGS2, ST6GALNAC5 and HBEGF expression, and with the transmigration ability of breast cancer (BC) cells.

Correlation Coefficient	miR-101-3p Expression
**PTGS2 expression**	r = −0.8059
**ST6GALNAC5 expression**	r = −0.7150
**HBEGF expression**	r = −0.9289
**Transmigrated cells**	r = −0.8756

miR-101-3p expression varies inversely compared to PTGS2, ST6GALNAC5 and HBEGF mRNA expression in BC cells and with the transmigration ability of BC cells (correlation coefficient r = −0.8756, −0.8059, −0.7150, and −0.9289 respectively).
